# The insertion/deletion (I/D) polymorphism in the *Angiotensin-converting enzyme *gene and cancer risk: a meta-analysis

**DOI:** 10.1186/1471-2350-12-159

**Published:** 2011-12-12

**Authors:** Yonggang Zhang, Jie He, Yao Deng, Jie Zhang, Xiaobo Li, Zhangpeng Xiang, Honglang Huang, Can Tian, Jin Huang, Hong Fan

**Affiliations:** 1 Department of Respiratory Medicine, West China Hospital, Sichuan University, Chengdu, Sichuan, 610041, China; 2 West China Medical School, Sichuan University, Chengdu, Sichuan, 610041, China; 3 Department of Laboratory Medicine, West China Hospital, Sichuan University, Chengdu, Sichuan 610041, China; 4 Key Laboratory of Laboratory Medicine, Ministry of Education, Zhejiang Provincial Key Laboratory of Medical Genetics, Wenzhou Medical College, Wenzhou, Zhejiang, 325035, China; 5 Department of Respiratory Medicine, The 452nd Military Hospital of China, Chengdu, Sichuan 610041, China

**Keywords:** Angiotensin-converting enzyme, cancer, meta-analysis, polymorphism, risk

## Abstract

**Background:**

The insertion/deletion (I/D) polymorphism in the *Angiotensin-converting enzyme *(*ACE*) gene has been implicated in susceptibility to cancer, but a large number of studies have reported inconclusive results. The aim of this study is to assess the association between the I/D polymorphism in the *ACE *gene and cancer risk by meta-analysis.

**Methods:**

A search was performed in Pubmed database, Embase database, Chinese Biomedical (CBM) database, China National Knowledge Infrastructure (CNKI) database and Weipu database, covering all studies until August 31, 2010. Statistical analysis was performed by using Revman4.2 and STATA 10.0.

**Results:**

A total of 25 case-control studies comprising 3914 cancer patients and 11391 controls were identified. No significant association was found between the I/D polymorphism and over all cancer risks (OR = 0.88, 95%CI = 0.73-1.06, P = 0.17 for DD+DI vs. II). In the subgroup analysis by ethnicity, no significant association was found among Asians and Europeans for the comparison of DD+DI vs. II. In the subgroup analysis by cancer types, no significant associations were found among lung cancer, breast cancer, prostate cancer, colorectal cancer, gastric cancer for the comparison of DD+DI vs. II. Results from other comparative genetic models also indicated the lack of associations between this polymorphism and cancer risks.

**Conclusions:**

This meta-analysis suggested that the *ACE *D/I polymorphism might not contribute to the risk of cancer.

## Background

The angiotensin-converting enzyme (ACE), a key enzyme in the renin-angiotensin system, plays the important roles of regulating of blood pressure and serum electrolytes [1,2]. It has also been implicated in the pathogenesis of several cancers, such as lung cancer, breast cancer, prostate cancer, gastric cancer and oral cancer [2-6]. It is differentially expressed in several carcinomas and may affect tumor cell proliferation, migration, angiogenesis, and metastatic behaviors [7]. Inhibition of ACE activity suppresses tumor growth and angiogenesis in vitro and vivo of animal models; moreover, epidemiologic studies have also indicated that ACE inhibitors might decrease the risk and mortality rate of cancers [2,7].

The human *ACE *gene is located on chromosome 17q23, and many polymorphisms have been identified [8]. The most widely studied polymorphism Insertion/Deletion (I/D, rs4646994) is located on intron 16[6]. It is characterized by the presence or absence of a 287-bp Alu repetitive sequence, which results in three genotypes: II, DI and DD [6]. The I/D polymorphism accounts for 20% to 50% of the variance in *ACE *expression or activity in blood and tissues among individuals [7]. Homozygote II may display as low as half of the plasma ACE level compared to the homozygote DD, whereas the heterozygote DI displays an intermediate level[2]. Recently, many studies investigated the role of this polymorphism in the etiology of cancers among various organs, including lung, breast, prostate, gastric, oral and others [3-7,9-18]. However, the observed associations of these studies were inconsistent, and a single study might be insufficient to detect a possible small effect of the polymorphism on cancers. Meta-analysis is a useful method for investigating the associations between diseases and risk factors because it uses a quantitative approach to combine the results of different studies on the same topic, potentially providing more reliable conclusions [19,20]. Considering the extensive role of ACE in the pathogenesis of cancers, a meta-analysis was performed on all eligible case-control studies to estimate the association between this polymorphism and cancer risks.

## Methods

### Publication search

We searched literatures in Pubmed database, Embase database, Chinese Biomedical database(CBM) database, Chinese National Knowledge Infrastructure(CNKI) database and Weipu database to identify articles that evaluated the associations between polymorphisms in *ACE *gene and cancer risks(Last search was updated on Aug 31st, 2010). The search terms were used as follows: '*cancer *or *carcinoma*' and '*ACE *or *angiotensin-converting enzyme*' in combination with '*polymorphism *or *mutation *or *variant'*. The languages were limited to English and Chinese. The following inclusion criteria were used in the meta-analysis: (1) the study should evaluate the I/D polymorphism in *ACE *gene and cancer risk, (2) the study should be a case-control design, (3) enough information had to be provided to calculate the odds ratio (OR) with 95% confidence interval (CI), (4) the distribution of genotypes in the control groups should be consistent with Hardy-Weinberg equilibrium (HWE). Accordingly, the following exclusion criteria were also used: (1) abstracts and reviews, (2) studies in which the genotype frequencies were not reported, (3) repeated or overlapped publications. For studies with the same case series by the same authors, the most recently published studies or studies with the largest numbers of subjects were included.

### Data extraction

Data were independently checked and extracted by two investigators. The following items were collected from each study: first author's name, year of publication, country of origin, ethnicity, genotyping methods, cancer type, total number of cases and controls, genotype distributions in cases and controls.

### Statistical analysis

For each case-control study, the HWE of genotypes in the control group was assessed by using Person's *X^2 ^*test. OR and 95% CI was used to assess the strength of the association between the I/D polymorphism and cancer risk. We calculated the OR and respective 95% CI by comparing the carriers of rare alleles with the wild homozygotes (DI+DD vs. II).

Heterogeneity among studies was assessed by a *X^2 ^*based *Q- *and *I^2^- *statistic. Heterogeneity was considered significant for *P *less than 0.10. The fixed-effects model and random-effects model were used to pool the results. When the *P *value of heterogeneity was greater than 0.10, the fixed-effects model was used, otherwise, the random-effects model was used, as it is more appropriate when heterogeneity is present. The significance of the pooled OR was determined by the *Z*-test and *P *less than 0.05 was considered as statistically significant. To evaluate the ethnicity-specific, cancer type-specific effects, subgroup analyses were performed by ethnic group ('*European*', '*Asian*', '*African-American' and 'Latino'*) and cancer types. Subgroup analyses by ethnicity were preformed if one ethnic population included more than three case-control studies. Subgroup analysis by cancer type were preformed if one cancer type canteined three and more than three individual studies. Comparisons of other genetic models were also performed (DD vs. DI+II, DD vs. II, DI vs. II and D vs. I).

Publication bias was investigated by using several methods. Visual inspection of asymmetry in funnel plots was carried out. The Begg's funnel plots and Egger's test were also used to statistically assess publication bias [21,22]. Sensitivity analysis was performed to assess the stability of the results by sequentially excluding each study [23]. All analyses were performed using the software Revman4.2 and STATA 10.0.

## Results

### Studies selection and characteristics in the meta-analysis

**Figure 1:**
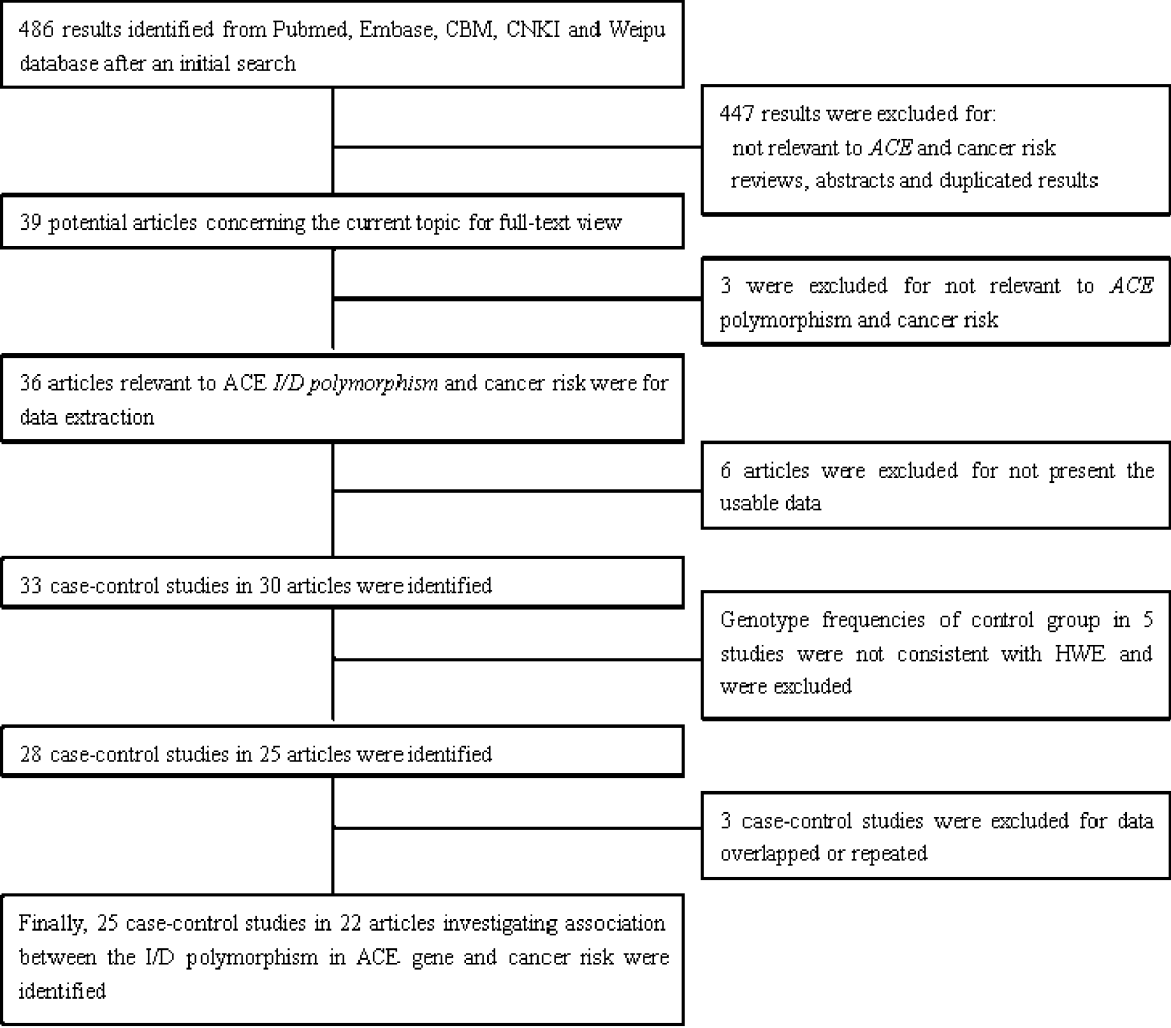
**Flow diagram of included/excluded studies**.

There were 486 results relevant to the search words in the selected databases (Figure 1). After reading the titles and abstracts, 39 potential articles were included for full-text view. Further screening of these articles, three of them were excluded for being not relevant to cancer risk with *ACE *gene polymorphism. Thus, 36 articles were left for data extraction. Six articles were excluded for not reporting the usable data. One article reported four cohorts each and each cohort was considered as a separate case-control study [6]. Thus, a total of 33 case-control studies in 30 articles were identified. Additionally, 5 case-control studies were excluded for the genotypes in control group not consistent with HWE[24-28], and 3 case-control studies were excluded for data overlapped or duplicated. Thus, a total of 25 case-control studies in 22 articles were finally identified [3-7,9,15-18,29,14,35]. The characteristics of included case-control studies are summarized in Table 1. Genotype and allele distributions for each case-control study are shown in Table 2. There were 8 studies of Asians [4,6,10,11,14,29,30,33], 14 of Europeans [3,5-7,9,12,13,15-17,31,32,34,35], 2 of Latinos [6,18], 1 of African-Americans [6]. In this meta-analysis, the most studied cancers were lung cancer and breast cancer, the genotype methods are a classic PCR assays

**Table 1:** Characteristics of case-control studies included in meta-analysis

Author	Year	Country	Ethnicity	Cancer type	No.(Cases/Controls)	Genotyping method
Arima, H[14]	2006	Japan	Asian	Cancers	176/761	PCR
Bardi, E[34]	2005	Hungary	European	Cancers	207/144	PCR
Cheon, K T[29]	2000	Korea	Asian	Lung	218/121	PCR
Ding, × J[30]	2008	China	Asian	Lung	121/33	PCR
Goto, Y[10]	2005	Japan	Asian	Gastric	202/454	PCR
Haiman, C A(AF)[6]	2003	USA	African-American	Breast	257/631	PCR
Haiman, C A(JP)[6]	2003	USA	Asian	Breast	284/357	PCR
Haiman, C A(Latinas)[6]	2003	USA	Latino	Breast	249/652	PCR
Haiman, C A(Whites)[6]	2003	USA	European	Breast	292/402	PCR
Holla, L[35]	1998	Czech	European	Leukemia	25/202	PCR
Nacak, M[3]	2010	Turkey	European	Lung	125/165	PCR
Nikiteas, N[16]	2007	Greece	European	colorectal	92/102	PCR
Rocken, C[7]	2005	Germany	European	Gastric	113/189	PCR
Rocken, C[31]	2007	Germany	European	Colorectal	141/189	PCR
Sierra, Diaz E[18]	2009	Mexico	Latino	Prostate	19/28	PCR
Sugimoto, M[4]	2006	Japan	Asian	Gastric	119/132	PCR
Toma, M[13]	2009	Romanian	European	Colorectal	108/150	PCR
Tunny, T J[17]	1996	Australia	European	Aldosterone-producing adenoma	55/80	PCR
Vairaktaris, E[32]	2009	Greece	European	Oral	160/153	PCR
van der Knaap, R[12]	2008	Netherlands	European	Colorectal, lung, breast, prostate	655/6015	PCR
Vaskù, V[9]	2004	Czech	European	T-cell lymphoma	77/203	PCR
Wang, H W[11]	2000	China	Asian	Lung	34/38	PCR
Yeren, A[15]	2008	Turkey	European	Lung	75/85	PCR
Yigit, B[5]	2007	Turkey	European	Prostate	48/51	PCR
Zhang Q Z[33]	2005	China	Asian	Lung	47/54	PCR

**Table 2:** Distribution of *ACE *genotype and allele among asthma patients and controls

Author	Case			Control			Case		Control		HWE for control population	
	
	II	ID	DD	II	DI	DD	I	D	I	D	X2	P
Arima, H[14]	65	87	24	295	372	94	217	135	962	560	1.978	0.160
Bardi, E[34]	74	89	44	52	71	21	237	177	175	113	0.166	0.683
Cheon, K T[29]	72	116	30	48	50	23	260	176	146	96	2.261	0.132
Ding, × J[30]	55	56	10	19	10	4	166	76	48	18	1.840	0.175
Goto, Y[10]	76	98	28	209	189	56	250	154	607	301	1.674	0.196
Haiman, C A(AF)[6]	62	118	77	100	310	221	242	272	510	752	0.254	0.614
Haiman, C A(JP)[6]	119	128	37	154	160	43	366	202	468	246	0.021	0.884
Haiman, C A(Latinas)[6]	73	127	49	189	301	162	273	225	679	625	3.677	0.055
Haiman, C A(Whites)[6]	79	129	84	91	187	124	287	297	369	435	1.613	0.204
Holla, L[35]	25	11	4	40	86	76	61	19	166	238	2.937	0.087
Nacak, M[3]	37	50	38	29	72	64	124	126	130	200	1.225	0.268
Nikiteas, N[16]	15	50	27	6	52	44	80	104	64	140	3.451	0.063
Rocken, C[7]	24	57	32	41	95	53	105	121	177	201	0.017	0.898
Rocken, C[31]	37	69	35	41	95	53	143	139	177	201	0.017	0.898
Sierra, Diaz E[18]	0	7	12	9	12	7	7	31	30	26	0.537	0.464
Sugimoto, M[4]	54	53	12	50	60	22	161	77	160	104	0.305	0.581
Toma, M[13]	25	50	33	30	73	47	100	116	133	167	0.029	0.864
Tunny, T J[17]	16	25	14	24	34	22	57	53	82	78	1.787	0.181
Vairaktaris, E[32]	30	70	60	9	66	78	130	190	84	222	1.054	0.305
van der Knaap, R[12]	141	329	185	1332	3006	1677	611	699	5670	6360	0.047	0.828
Vaskù, V[9]	19	37	21	43	103	57	75	79	189	217	0.078	0.780
Wang, H W[11]	10	6	18	13	18	7	26	42	44	32	0.031	0.861
Yeren, A[15]	4	39	32	14	37	34	47	103	65	105	0.522	0.470
Yigit, B[5]	4	19	25	12	24	15	27	69	48	54	0.157	0.692
Zhang Q Z[33]	21	21	5	20	30	4	63	31	70	38	2.567	0.109

### Meta-analysis results

**Figure 2:**
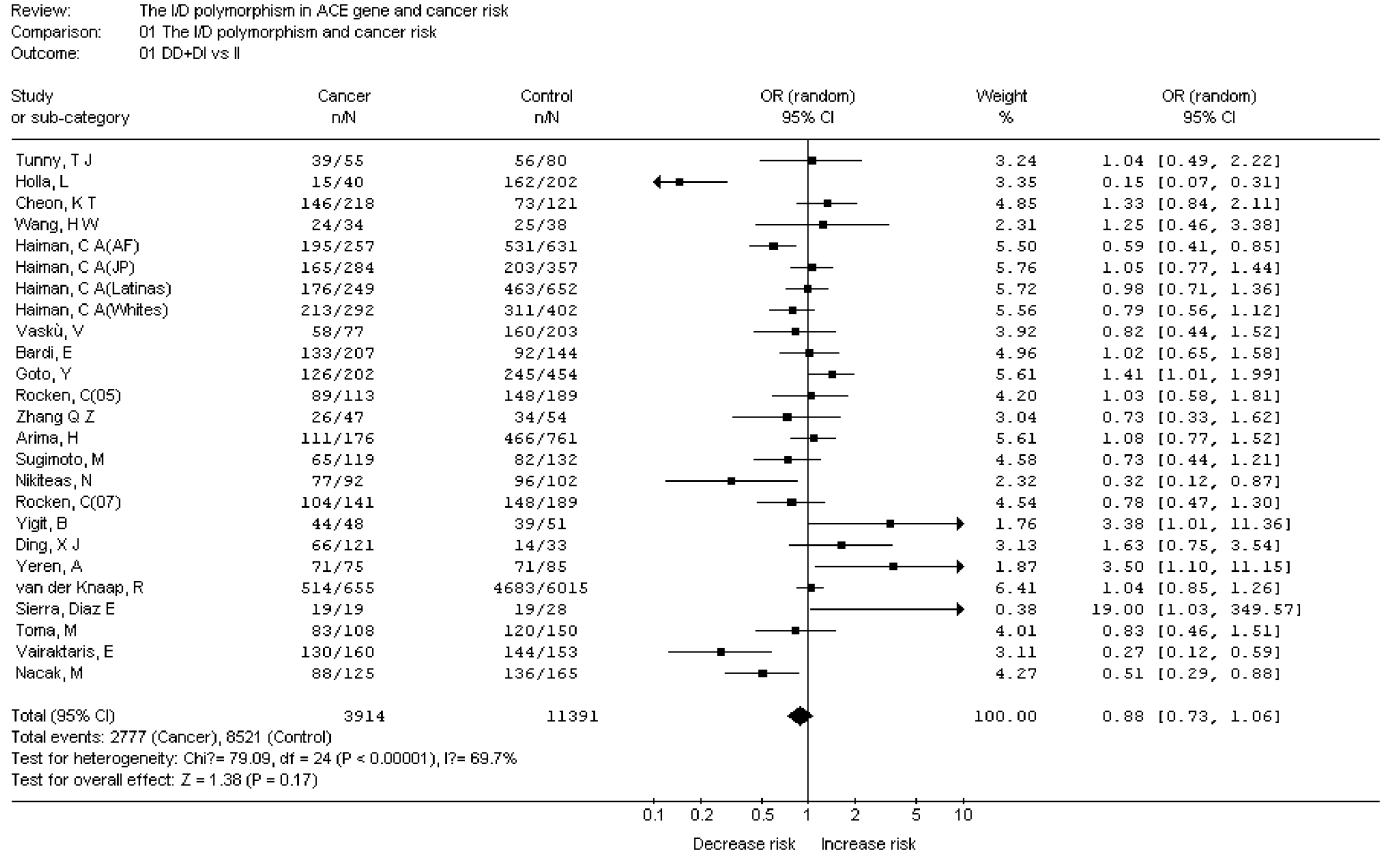
**Meta-analysis with a random-effects model for the association between cancer risk and the *ACE *I/D polymorphism (DD+DI vs II)**.

As shown in Figure 2, heterogeneity of DD+DI vs. II for all studies was analyzed and the value of *X^2 ^*was 79.09 with 24 degrees of freedom and *P *< 0.00001 in a random-effects model. Additionally, I-square value is another index of the test of heterogeneity. In Figure 2, the I-square was 69.7%, suggesting the presence of heterogeneity. Thus, the random-effects model was chosen to synthesize the data. OR was 0.88(95%CI = 0.73-1.06) and the test for overall effect *Z *value was 1.38 (*P *= 0.17). The results suggested that the variant D allele carriers (DI+DD) do not have a significant increased risk of cancer compared with those individuals without D allele (II). Summary of the results of other genetic comparisons are listed in Table 3.

**Table 3:** Summary of different comparative results

Variables	N	Cases/Controls	DD+DI vs II		DD vs DI+II		DD vs II		D vs I		DI vs II	
			
			OR(95%CI)	P*	OR(95%CI)	P*	OR(95%CI)	P*	OR(95%CI)	P*	OR(95%CI)	P*
Total	25	3914/11391	0.88(0.73, 1.06)	0.38	0.93(0.80, 1.09)	0.39	0.90(0.73, 1.12)	0.34	0.94(0.83, 1.07)	0.34	0.89(0.75, 1.06)	0.20
Subgroup by ethnicity												
Asian	8	1201/1950	1.13(0.96, 1.33)	0.15	1.05(0.74, 1.49)	0.78	1.09(0.83, 1.44)	0.53	1.08(0.93, 1.25)	0.34	0.85(0.68, 1.06)	0.14
European	14	2188/8130	0.89(0.73, 1.09)	0.26	0.89(0.73, 1.09)	0.26	0.76(0.53, 1.08)	0.12	0.86(0.71, 1.03)	0.10	0.78(0.60, 1.01)	0.06
Subgroup by cancer type												
Lung	7	737/6511	1.04(0.70, 1.55)	0.85	0.99(0.67, 1.48)	0.98	1.02(0.62, 1.69)	0.93	1.03(0.80, 1.31)	0.83	1.01(0.64, 1.61)	0.95
Breast	5	1235/8057	0.88(0.71, 1.09)	0.24	0.95(0.75, 1.21)	0.70	0.86(0.64, 1.16)	0.33	0.94(0.81, 1.09)	0.41	0.88(0.72, 1.09)	0.24
Colorectal	4	517/6456	0.81(0.53, 1.24)	0.34	0.83(0.66, 1.05)	0.11	0.73(0.45, 1.18)	0.20	0.87(0.71, 1.06)	0.17	0.87(0.58, 1.31)	0.50
Gastric	3	434/775	1.06(0.71, 1.59)	0.78	0.94(0.66, 1.36)	0.76	0.96(0.55, 1.65)	0.87	1.00(0.74, 1.34)	1.00	1.12(0.79, 1.58)	0.52
Prostate	3	276/6094	2.44(0.66, 8.97)	0.18	2.05(0.74, 5.62)	0.17	3.48(0.63, 19.13)	0.15	2.05(0.84, 5.02)	0.11	1.66(0.67, 4.07)	0.27

*: P value for Q-test.

**Figure 3:**
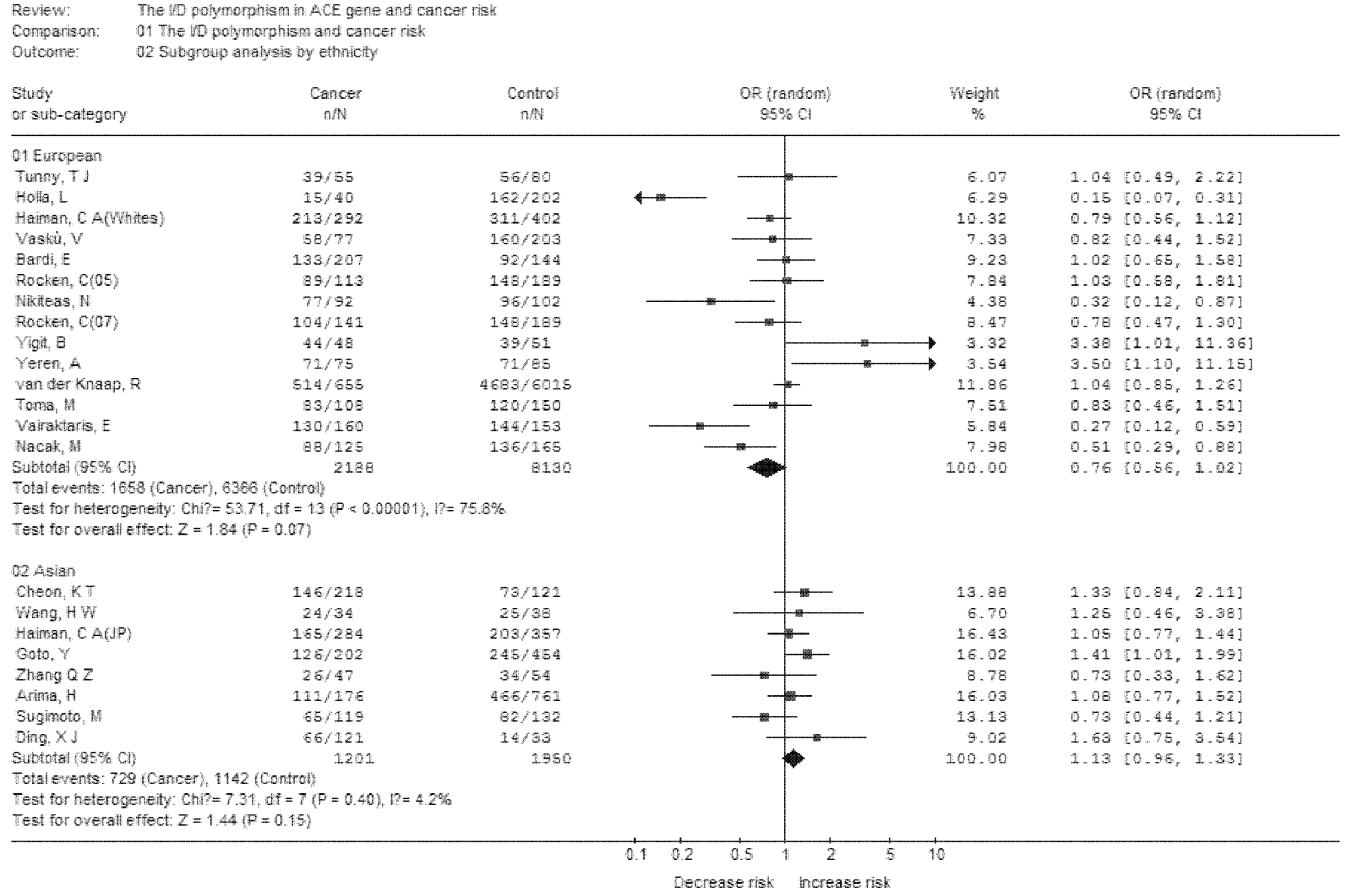
**Meta-analysis with a random-effects model for the association between cancer risk and the *ACE *I/D polymorphism (DD+DI vs II): subgroup analysis by ethnicity**.

**Figure 4:**
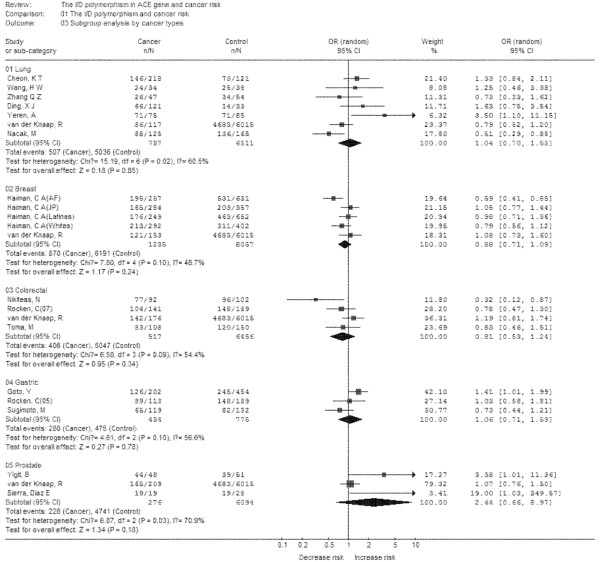
**Meta-analysis with a random-effects model for the association between cancer risk and the *ACE *I/D polymorphism (DD+DI vs II): subgroup analysis by cancer type**.

Subgroup analyses were performed after stratifications of the data by ethnicity and cancer types. In the subgroup analysis by ethnicity (Figure 3), no significant increased risks were found in *Europeans *and *Asians*. In the subgroup analysis by cancer types (Figure 4), no significant increased risk was found in lung cancer, breast cancer, colorectal cancer, gastric cancer, prostate cancer. Thus, the polymorphism may not increase cancer risk in different population and different cancers.

### Publication bias

**Figure 5:**
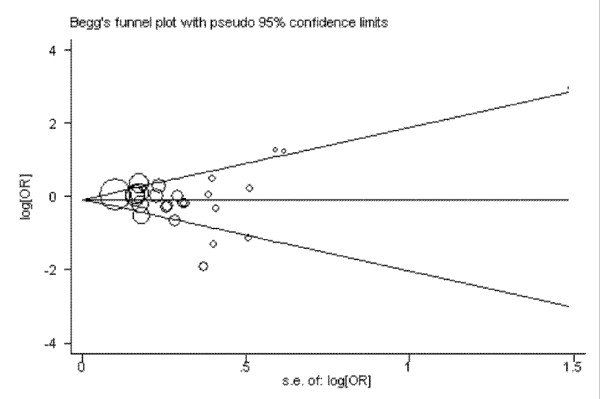
**Begg's funnel plot for publication bias in selection of studies on the *ACE *I/D polymorphism (DD+DI vs II)**.

Begg's funnel plot and Egger's test were performed to assess the publication bias of the literatures. The shape of the funnel plots seemed approximately symmetrical (DD+DI vs. II) and the Egger's test did not show any evidence of publication bias (*t *= 0.45 and *P *= 0.655 for DD+DI vs. II) (Figure 5).

### Sensitivity analysis

Sensitivity analysis was analyzed as previous study [23]. Briefly, after excluding each case-control study for DD+DI vs. II comparative (Table 4), statistically similar results were obtained, suggesting the results of this meta-analysis are stable.

**Table 4:** Sensitivity analysis for the current meta-analysis: ORs with 95%CIs and P value were results after excluding each case-control study for DD+DI vs.II.

Author	OR	95%CT	P
Arima, H[14]	0.87	0.71-1.06	0.16
Bardi, E[34]	0.87	0.72-1.06	0.16
Cheon, K T[29]	0.86	0.71-1.04	0.12
Ding, × J[30]	0.86	0.71-1.04	0.12
Goto, Y[10]	0.85	0.71-1.03	0.10
Haiman, C A(AF)[6]	0.90	0.74-1.09	0.27
Haiman, C A(JP)[6]	0.87	0.71-1.06	0.16
Haiman, C A(Latinas)[6]	0.87	0.71-1.06	0.17
Haiman, C A(Whites)[6]	0.88	0.78-1.07	0.21
Holla, L[35]	0.93	0.79-1.09	0.38
Nacak, M[3]	0.90	0.75-1.09	0.27
Nikiteas, N[16]	0.90	0.75-1.08	0.26
Rocken, C[7]	0.87	0.72-1.06	0.16
Rocken, C[31]	0.88	0.73-1.07	0.21
Sierra, Diaz E[18]	0.87	0.72-1.04	0.13
Sugimoto, M[4]	0.89	0.73-1.07	0.22
Toma, M[13]	0.88	0.73-1.07	0.19
Tunny, T J[17]	0.87	0.72-1.06	0.16
Vairaktaris, E[32]	0.91	0.76-1.09	0.31
van der Knaap, R[12]	0.87	0.71-1.07	0.18
Vaskù, V[9]	0.88	0.73-1.07	0.19
Wang, H W[11]	0.87	0.72-1.05	0.15
Yeren, A[15]	0.86	0.71-1.03	0.10
Yigit, B[5]	0.86	0.71-1.03	0.10
Zhang Q Z[33]	0.88	0.73-1.07	0.20

## Discussion

ACE is a key enzyme in the renin-angiotensin system, which is involved in the regulation of blood pressure and serum electrolytes. In recent years, more evidences indicated that the enzyme was associated with the pathogenesis of cancers. It may influence tumor cell proliferation, migration, angiogenesis and metastatic behaviors. Given the important roles of ACE in cancer etiology, it is possible that genetic variations of the *ACE *gene may modulate the risk of cancer. The I/D polymorphism in intron 16 of the *ACE *gene is the most extensively studied polymorphism. This polymorphism is based on insertion or deletion of a 287-bp Alu sequence, leading to a change in the plasma ACE level. Growing number of studies have been published to investigate the associations between this polymorphism with cancer risk; however, the results were inconsistent and conflict. In order to resolve this issue, we conducted a meta-analysis of 25 case-control studies, including 3914 cases and 11391 controls, to evaluate the associations between the *ACE *I/D polymorphism and cancer risks.

Our results showed that the *ACE *I/D polymorphism was not associated with cancer risks. Moreover, in other comparative genetic models, no significant associations were found in any genetic models. These results indicated that this polymorphism may not contribute to cancer risks. Although previous studies revealed possible roles of ACE in cancer etiology, our result suggested that these roles may not account by the variant of *ACE *gene. Although the exact mechanism of this enzyme in cancer etiology is not so clear, our results may indicate that ACE I/D polymorphism may not influence cancer risk. In addition, considering the possible role of this polymorphism in serum ACE level, it is possible that the cancer risk may be modified by ACE level, but not by the variant. Thus, future studies are warranted to identify the associations between *ACE *polymorphism, ACE levels and cancer risk.

Considering the property of genetic background may affect the results of genetic association studies, we performed subgroup analysis by ethnicity. Two subgroups were included in this meta-analysis: 'European' and 'Asian'. In this meta-analysis, we didn't find a significant association between this polymorphism and cancer risk in any sub-populations. Moreover, no significant associations were found in any other genetic models. Interestingly, this polymorphism and cancer risk in Asians and Europeans were all inversely associated, although they were not statistically significant. These results may suggest that this polymorphism may exert varying effects in different populations. However, all included studies were from European, Asian, African-American and Latino populations, further studies are necessary to validate these findings for other ethnic populations, especially in Africans.

In another subgroup analysis by cancer types, we found that this polymorphism is not associated with increased risks in all sub-cancer types (lung, breast, prostate, gastric and colorectal). Considering the limitation of studies in each subgroup, the weak associations between this polymorphism and different cancers should be discussed. It is possible that these null associations may be due to chance because studies with small sample size may have insufficient statistical power to detect a slight effect. Considering the limited studies in each cancer type, our results should be explained with caution.

Heterogeneity is one of the important issues when performing meta-analysis. We found that heterogeneity between studies existed in overall comparisons. After subgroup analysis by ethnicity and cancer types, the heterogeneity was effectively decreased or removed in Asians and some cancer types, suggesting that certain effects of genetic variants are cancer specific and ethnic specific. The stability of this meta-analysis was analyzed by sequentially excluding individual studies; our results indicated stability of results. Publication bias is another important issue which should also be discussed in meta-analysis. After evaluating the publication bias by Egger's funnel plots and Begg's test, we did not detect a publication bias, indicating the strength of the results.

Cancers have been considered as genetic diseases, and many genetic variants are contributed to cancer risks [36-40]. It is worthy to mention the recently studies of genetic analysis by genome wide-association studies (GWAS). Up to now, a large number of GWAS for cancers have been published, and a large number of cancer susceptible loci were found [36-38,41-46]. However, none of them indicated the *ACE *D/I polymorphism, which can be partly explained by the chips which were used in the original studies. There is probably a lack of adequate coverage on chips which also identify insertion or deletion a 287-bp sequence. It is noted worthy, the SNP rs4343, which is considered as a good proxy in Caucasians for I/D variant (r2 > 0.80), have been studied by Illumina genotyping arrays [47,48]. Thus, further studies may investigate rs4343 for cancer risk by GWAS to help to resolve whether the I/D polymorphism is associated with cancer risk.

We have to mention a recently published study by Ruiter in 2011[49]. They also investigated the ACE I/D polymorphism with the risk of cancers. There were some differences between these two studies. First, the current meta-analysis included more case-control studies than Ruiter's study. Second, some issues which may affect the results of meta-analysis were addressed in our study, such as publication bias, sensitivity analysis and HWE analysis. Third, the current study is a meta-analysis, and Ruiter's study is more like a review. Despite of these differences, our study also indicated the ACE I/D polymorphism might not contribute to the risk of cancer, which is consistent with Ruiter's study.

Some limitations of this study should be addressed. First, only published studies in Chinese and English which were included by the selected databases were included for data analysis, some potential studies which were included by other databases or published with other languages or unpublished could be missed. Second, due to lack of original data, we could not evaluate the potential interactions of gene-gene and gene-environment. Third, this meta-analysis included data from Europeans, Asians, African-American and Latino populations, so that, the results are applicable to only these ethnic groups. Fourth, it is reported that PCR amplification of ACE I/D polymorphism using only flanking primer pairs would misclassify 4-5% of the ID genotype as the DD genotype and a second PCR should performed to confirm the DD genotype[50]. However, only a small portion of included studies performed a second PCR, indicating the possibility of imprecise results of the meta-analysis. Fifth, some other important factors may also bias our results, such as smoking status, the heterogeneity of cancer patients (pre- or post -menopausal breast cancer patients); genotyping technique changes over time and other unknown function of RAS system et al. However, this meta-analysis also has some advantages. First, the comprehensive meta-analysis included more than 15,000 individuals; it is statistically more powerful than any single study. Second, this is a comprehensive meta-analysis concerning cancer risk and *ACE *D/I polymorphism and the result also indicated a gene in RAS may not contribute to cancer risk. Third, the result of publication bias is not significant; indicating the conclusion of this study may be unbiased.

In summary, this meta-analysis suggests that the I/D polymorphism in the *ACE *gene may not contribute to susceptibility to cancer. However, larger well-designed studies are warranted to validate these findings. Moreover, future studies should also investigate gene-gene and gene-environment interactions to better display the association between the polymorphisms in *ACE *gene and cancer risk.

## Conclusion

These results suggest that the D/I polymorphism in *ACE *gene may not contribute to susceptibility to cancer.

## Conflict of interests

The authors declare that they have no competing interests.

## Authors' contributions

HF designed the research. YGZ and JZ searched the publications, extracted the data and wrote the materials and methods, results, discussion. YD checked all data. ZPX, HLH and CT was responsible for data synthesis and helped designed the study's analytic strategy. JH wrote the introduction. JH and XBL edited the manuscript. All authors read and approved the final manuscript.

## Pre-publication history

The pre-publication history for this paper can be accessed here:

http://www.biomedcentral.com/1471-2350/12/159/prepub
